# Factors associated with traction alopecia in women living in Yaoundé (Cameroon)

**DOI:** 10.1186/s12905-023-02716-2

**Published:** 2023-11-08

**Authors:** Letitia Fiona Mbussuh Nzeng, Georges Nguefack-Tsague, Dahlia Noëlle Tounouga, Mohamed Maciré Soumah, Franck Albert Armand Mbang, Odette Berline Sigha, Grace Anita Nkoro, Rose Kotto, Emmanuel Armand Kouotou

**Affiliations:** 1 Faculty of Medicine and Biomedical Sciences, University of Yaoundé 1, Yaoundé, Cameroon; 2 National University Teaching Hospital of Cotonou, 01 PO Box 386, Cotonou, Benin; 3 University Teaching Hospital of Conakry, Gamal Abdel Nasser University of Conakry, Conakry, Guinea; 4 Faculty of Health Sciences, University of Bamenda, Bambili, Cameroon; 5 Faculty of Medicine and Pharmaceutic Sciences, University of Douala, Douala, Cameroon

**Keywords:** Traction alopecia, Hairstyles, Associated factors, Women, Yaoundé

## Abstract

**Background:**

Traction alopecia (TA) is very common in Africa but few studies on African population are available. We sought to determine factors associated with TA and measure the association between these factors and TA.

**Methods:**

We carried out an analytical cross-sectional study in 29 hairdressing saloons in the city of Yaoundé. A questionnaire was administered and scalp exams were performed in order to look for TA and determine Marginal TA severity score if present. Participants were separated in two groups: TA group and a group without TA.

**Results:**

We finally included 223 women (77 having TA and 146 without TA). The median age was 26 years for women with TA and 24 years for women without TA. The factors associated with traction alopecia we found included: age ≥ 35 years (adjusted OR = 4; *p* = 0.016). Hairdressing undertaken by hairdressers only (adjusted OR = 0.2; *p* = 0.008), the avoidance of the regular use of nets, caps and head ties (OR = 0.2; *p* = 0.006) and relaxing hairs once a year or less (adjusted OR = 0.2; *p* = 0.005) could be protective factors. As well, we found a positive correlation between age and TA severity (*r* = 0.235; *p* < 0.001).

**Conclusion:**

Age and some haircare practices are associated to TA occurrence in our context. Women therefore need to be educated on these various factors that could be able to cause, worsen or prevent TA.

## Background

Alopecia is defined as a partial or total hair loss whatever the cause. This pathology can occur in individuals of all ages, races or genders. Alopecia represents a frequent complaint in dermatology clinic [1]. The main mechanisms leading to alopecia are : hair shaft defects, alteration of the hair shaft’s cycle (telogen effluvium, anagen arrest), destruction of hair follicles and miniaturization of the follicle [2].

Alopecia is broadly classified in two groups: scarring (i.e., cicatricial) and nonscarring alopecia. Scarring alopecia are characterized by an irreversible destruction of the hair follicle. Scarring alopecia can result from a primary disease of the hair follicle (primary alopecia) or can be secondary to another process (secondary alopecia) [3, 4]. Nonscarring alopecia do not affect the hair follicle. They are divided into traumatic and nontraumatic alopecia. Traumatic alopecia include: compression, compulsion, twitch alopecia and traction alopecia (TA) [5].

TA is localised traumatic nonscarring alopecia which affects almost exclusively people of African descent [6]. TA in absence of treatment evolves to definitive hair loss. The main factors associated with TA found in literature are : old age, the regular use of traumatic hairstyles (ponytails, tight braids, extension use…), hairdressing symptoms (pimples, crusts, pain), the combined use of hair dyes and chemical hair relaxers, heat processing of relaxed hair [6–8]. Afro hair (due to its characteristics) and chemical relaxation of the hair make them more susceptible to chronic mechanical trauma [6].

However, though TA is not fundamentally associated to morbidity, this pathology is responsible of an aesthetic prejudice. This prejudice induces a major psychological impact that alters considerably the quality of life of these patients [1, 9]. Therefore, prevention is crucial, based on factors which could induce, worsen or delay the onset of TA [10]. Yet, very few studies have been carried out on this topic, particularly in African individuals. Thus, this study aimed to determine factors associated with TA and measure the association between these factors and TA.

## Methods

### Design and site of the study

We carried out a descriptive and analytic cross-sectional study during 7 weeks as from the 07th June to the 21st July 2020 in 29 hairdressing salons (23 classic saloons and 6 high class saloons) in the town of Yaoundé. Since we didn’t have an exact estimation of the number of hairdressing saloons present in the town of Yaoundé, saloons were chosen randomly in each of the 7 subdivisions in Yaoundé. A classic saloon/high class saloon ratio was not defined.

We have defined Classic saloon as a saloon which offers hairdressing as main service and which sells hair accessories; and High-class saloon as saloon offering in addition to hairdressing, body care, nail care, massage etc.

The minimal sample size was 171 participants and given by the formula below:

$$\mathbf{n}=\frac{{\mathbf{t}}^{2}\times \mathbf{p}\times (1-\mathbf{p})}{{\mathbf{m}}^{2}}$$

**N:** minimal sample size for statistically significant results.

**T:** 1.96 for a confidence interval of 95%.

**P:** traction alopecia’s prevalence in women 31.7% [11].

**m**^**2**^**:** standard error 0.07.

Our target population was women living in Yaoundé and our source population was made of women visiting hairdressing saloons in the town of Yaoundé. Women aged of at least 18 years present in a hairdressing saloon during our visit and that gave their informed consent were included in our study. Exclusion criteria were: an incomplete interview, hairdressing abstention for at least five years and hairstyles limiting the scalp margins visualisation.

### Data collection procedure

Before going on the field, ethical approval was obtained from the Institutional Committee for Ethics and Research of the Faculty of Medicine and Biomedical Sciences of The University of Yaoundé I. Subsequently, verbal authorizations were given by the administrative authorities of the various subdivisions as well as the different hairdressing saloon owners selected for our study.

We built a single team for data collection. Hairdressing saloons were visited one time. Once the team arrived in a hairdressing saloon, the object of the study was presented to every potential participant and the consent to participate to the study was requested. Then, a preconceived and pretested questionnaire was given to those who agreed to participate to our study. Each participant was filling the form under the supervision and with the help of the investigator. The questionnaire was globally made of sociodemographic data (age, profession, monthly income, native region) and the various haircare practices (grooming habits hair relaxing and hair hygiene). Then a scalp examination was performed inside the hairdressing saloon in a space allocated for this exercise. In order to achieve this, the examiner inspected the participants’ scalp and then palpated the hairs in order to appreciate their density. The scalp margins were separated in anterior and posterior margins by an imaginary line joining both tragi.

**Fig. 1:**
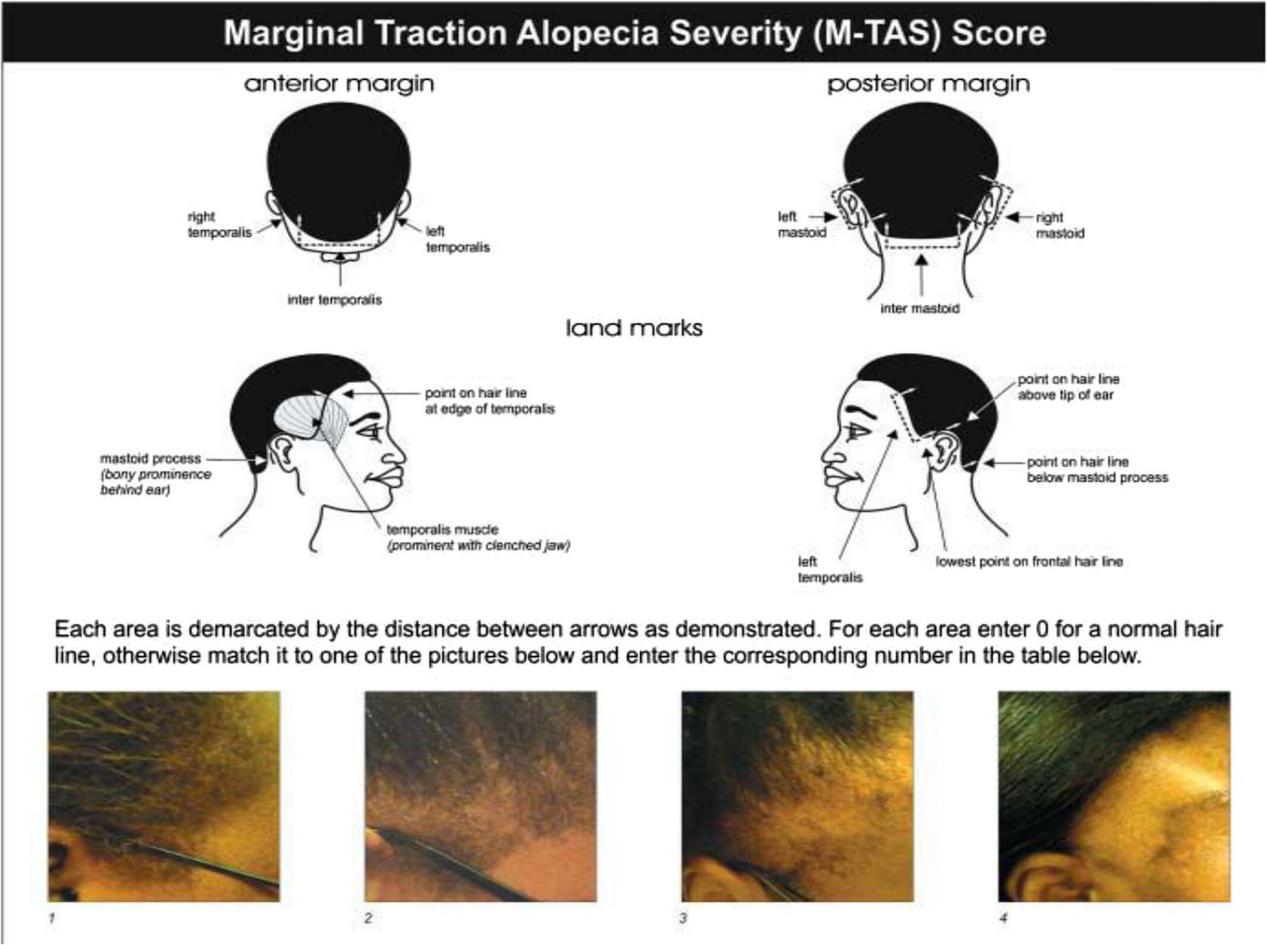
Tool for marginal traction alopecia score evaluation

As a reminder, the medial edges of the temporal muscles divide the anterior margin in three areas: left temporalis, right temporalis and inter temporalis. Temporal muscles were palpated easily by asking the participant to clench both jaws. The posterior margin is also divided in three areas: left mastoid, right mastoid and inter mastoid by both mastoid prominences [11]. Data relative to each region’s examination were directly reporter on the data collection form. The picture grid of the Marginal Traction Alopecia Severity Score (M-TAS) proposed by Khumalo et al. (Fig. 1) was used to research and evaluate TA [12]. When the examination was normal, the zero score was given meaning absence of TA. Otherwise, when alopecic areas or scarcity of hairs were detected, the severity was appreciated with the picture grid of the questionnaire in order to quote a score between 1 and 4. The score of each area was summed in order to obtain a total. The M-TAS ranges from zero to 24 and is interpreted as follows [11]:

- **M-TAS = 0**: no traction alopecia.
- **1 ≤ M-TAS ≤ 3**: mild traction alopecia.
- **4 ≤ M-TAS ≤ 6**: moderate traction alopecia.
- **M-TAS ˃ 6**: severe traction alopecia.

At the end of this process, a counselling was made to all the participants of our study on TA and preventive measures. As well, a counselling was made on the behavioural changes relative to hair hygiene and haircare.

### Data analysis

Data collected were digitized with the software Epi Info version 7.2.5 and analysed with the software IBM SPSS version 25. Quantitative data were represented by their median and interquartile range (IQR). The average ages of the group with TA and the group without TA were compared using the U Mann-Whitney test. Qualitative data were represented in terms of number and proportions. Qualitative data were compared with the Chi^2^ test or the Fischer exact test when the conditions for Chi^2^ test were not filled. A *p-value* < 0.05 was considered as statistically significant. A binomial logistic regression was used to research factors associated to TA. For analysis purposes, ages have been merged into age groups. The association was represented by the adjusted odds ratio and the 95% confidence interval. Data with a p-value < 0.2 in univariate analysis as well as variables acknowledged as favouring TA (hairstyle, hair relaxation frequency) were integrated in the multivariate model. For multivariate analysis, we used a step-by-step model in including at the beginning variables and excluding them progressively if *p* > 0.1 at each step. The association between the participants’ age and the M-TAS was evaluated with the Spearman correlation test. Tables were realised with the software Microsoft Office Excel version 2020.

### Ethical and administrative considerations

The research was carried out in the respect of the 4 fundamental principles of Helsinki declaration on human research. Before inclusion in the study, each participant was informed on the advantages and the constraint relative to our study. Each participant of the study gave her informed consent. Participation to our study was free and any refusal did not lead to consequences. Our research proposal was submitted and approved by the Institutional Committee for Ethics and Research of the Faculty of Medicine and Biomedical Sciences of the University of Yaoundé I for research approval and authorization (Reference No: 346/UY1/FMSB/VDRC/DAASR/CSD).

## Results

**Fig. 2:**
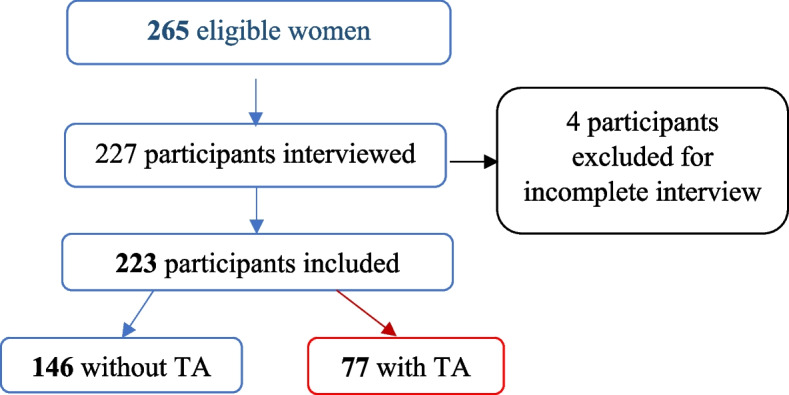
Flowchart of the participants enrolment

For this study, among 265 recruited, we finally included 223 participants; we identified 77 women with TA and 146 without TA (Fig. 2).

The median age was 24 years with an interquartile range [21; 27 years] and another range from 18 to 55 years. For the TA group, the median age was 26 years with an interquartile range [22; 33 years] and a range from 18 to 55 years. The median age for the group without TA was 24 years with an interquartile range [21; 26 years] and a range from 18 to 40 years. The most represented age group was the one of participants aged from 18 to 24 years (Table 1).

**Table 1:** Distribution of the sociodemographic characteristics in TA group and the group without TA

Characteristics *N* = 223	TA *n* = 77 (%)	No TA *n* = 146 (%)	*p*-value
**Age**			
**Mean**	28.5	24.3	
**Median [IQ]**	26 [22.5 ;33]	24 [21; 26]	**0.0001**
**Minimum**	18	18	
**Maximum**	55	45	
**Region of origin**			
**Farnorth/North/Adamoua**	2 (2.6)	4 (2.7)	
**Centre/South/East**	28 (36.4)	68 (46.6)	0.557
**Littoral/Southwest**	13 (16.9)	21 (14.4)	
**West/Northwest**	34 (44.1)	53 (36.3)	
**Level of education**			
**Primary**	8 (10.3)	3 (2.1)	
**Secondary**	16 (20.8)	15 (10.3)	**0.001**
**Higher**	53 (68.8)	128 (87.6)	
**Profession**			
**Hairdresser**	9 (11.7)	7 (4.8)	0.058
**Others**	68 (88.3)	139 (95.2)	

### Sociodemographic factors

The TA group was older than the group without TA with a statistically significant difference (*p* ˂ 0.008). Participants under 35 years old were mostly affected. Thus, the TA group had a lower level of education compared to that of the group without TA (*p* ˂ 0.001), (Table 1).

### Factors related to hair grooming habits

 All the women of the TA group used extensions to groom their hairs, a proportion statistically superior to the one of the disease-free women (*p* = 0.013), (Table 2). The proportion of women with TA which had their hairstyles done by a hairdresser or a family member / friend that didn’t have hairdressing training was statistically inferior to the proportion found in disease free women (*p* = 0.004). On the other hand, the regular use of nets, caps and head ties was less common in the group without TA compared to the TA group; this disparity was statistically significant (*p* = 0.036), (Table 2).

**Table 2:** Distribution of hair grooming habits among the TA group and the group without TA

Characteristics *N* = 223	TA *n* = 77 (%)	No TA *n* = 146 (%)	*p*-value
**Type of hairstyle**			
** Hairstyles with extension**	77 (100)	135 (92.5)	**0.013**
** Hairstyles without extension**	0 (0)	11 (7.5)	
**Hair grooming frequency**			
** < 3 weeks**	28 (36.4)	53 (36.3)	0.993
** ≥ 3 weeks**	49 (63.6)	93 (63.7)	
**Hair grooming provider**			
** Family member / friend who is not hairdresser**	6 (7.8)	17 (11.6)	
** Hairdresser**	59 (76.6)	124 (84.9)	**0.004**
** Both**	12 (15.6)	5 (3.4)	
**Wig cap use**			
** Yes**	42 (54.5)	89 (55.4)	0.355
** No**	35 (45.5)	57 (44.6)	
**Hairdressing related symptoms frequency**			
** Always**	8 (10.4)	12 (8.2)	
** Sometimes**	23 (29.9)	56 (38.4)	0.438
** Rarely/never**	46 (59.7)	78 (53.4)	
**Hair brushing**			
** Yes**	44 (57.2)	87 (59.6)	0.724
** No**	33 (42.8)	59 (40.4)	
**Head tie/cap/net use**			
** Yes**	72 (93.5)	122 (83.6)	
** No**	5 (6.5)	24 (16.4)	**0.036**
**Hair shaving**			
** Yes**	65 (84.4)	112 (76.7)	0.176

The age at the first use of extensions was similar in both groups (Table 3).

**Table 3:** Distribution of both groups according to the age at the first use of extensions

Characteristics *N* = 223	TA *n* = 77 (%)	No TA *n* = 146 (%)	*p*-value
**Age at the first use of extensions**			
** Before 10 years**	40 (53.4)	87 (59.6)	0.237
** After 10 years**	37 (46.6)	59 (40.4)	
**Age at the first wig use**			
** Before 15 years**	8 (10.4)	22 (15.1)	
** After 15 years**	69 (89.6)	124 (84.9)	0.365

### Factors related to chemical and thermal treatments

The proportion of women with TA that used chemical hair relaxers was statistically higher than in women without TA (*p* < 0.001) (Table 4).

**Table 4:** Factors related to hair treatment and hygiene

**Hair straightening process and use of chemical and thermal treatment in both groups**			
**Characteristics** ***N*** **= 223**	**TA** ***n*** **= 77 (%)**	**No TA** ***n*** **= 146 (%)**	***p*** **-value**
**Chemical hair relaxation**			
**Yes**	77 (100)	119 (81.5)	**< 0.001**
**No**	0 (0)	27 (18.5)	
**Age at the first hair relaxation**			
**Before 10 years**	16 (20.8)	20 (13.7)	0.172
**After 10 years**	61 (79.2)	126 (86.3)	
**Hair relaxation / year**			
**No more than once**	11 (14.2)	64 (43.8)	
**2–3 times**	38 (49.4)	60 (41.1)	**< 0.001**
**> 3 times**	28 (36.4)	22 (15.1)	
**Hair straightening provider**			
**Family member**	15 (19.5)	21 (14.4)	
**The participant**	9 (11.7)	9 (6.1)	**0.001**
**Hairdresser**	53 (68.8)	89 (61.0)	
**Nobody**	0 (0)	27 (18.5)	
**Burns after relaxation**			
**Never/Rarely**	41 (53.2)	82 (56.2)	
**Sometimes**	23 (30)	52 (35.6)	0.139
**Often/always**	13 (16.8)	12 (8.2)	
**Characteristics** ***N*** **= 223**	TA *n* = 77 (%)	No TA * n* = 146 (%)	*p*-value
**Hair dye use**			
**Yes**	32 (41.6)	50 (34.2)	0.282
**No**	45 (58.4)	96 (65.8)	
**Heat treatment**			
**Yes**	64 (88.3)	105 (71.9)	0.063
**No**	13 (16.9)	41 (28.1)	
**Hair hygiene of participants**			
**Characteristics** ***N*** **= 223**	**TA** ***n*** **= 77 (%)**	**No TA** ***n*** **= 146 (%)**	***p*** **-value**
**Hair washing/month**			
** No more than once**	39 (50.6)	81 (55.5)	0.492
** At least twice**	38 (49.4)	65 (44.5)	
**Hair washing product**			
** Shampoo**	55 (71.4)	113 (77.4)	0.326
** Others**	22 (28.6)	33 (22.6)	
**Hair oil**			
** Natural oil**	22 (28.6)	46 (31.5)	
** Manufactured oil**	23 (29.9)	26 (17.8)	0.203
** Both**	31 (40.2)	70 (47.9)	
** None**	1 (1.3)	4 (2.7)	

The age at the first chemical hair straightening was similar in both groups. Meanwhile, the annual frequency of hair relaxation was significantly higher in the TA group compared to the group without TA (*p* ˂ 0.001). The proportion of women with TA whose hair was relaxed by themselves or by a family member was statistically higher than in the group without TA (*p* = 0.001) (Table 4).

Women of the TA group used hair dyes, hairdryer and heat iron more often than the disease-free women but the difference was not significative (Table 4).

### Factors related to hair hygiene

The hair washing frequency was similar in both groups. The majority of participants irrespective of the group used shampoo to wash their hair (Table 4).

### Correlation between age and traction alopecia’s severity

**Fig. 3:**
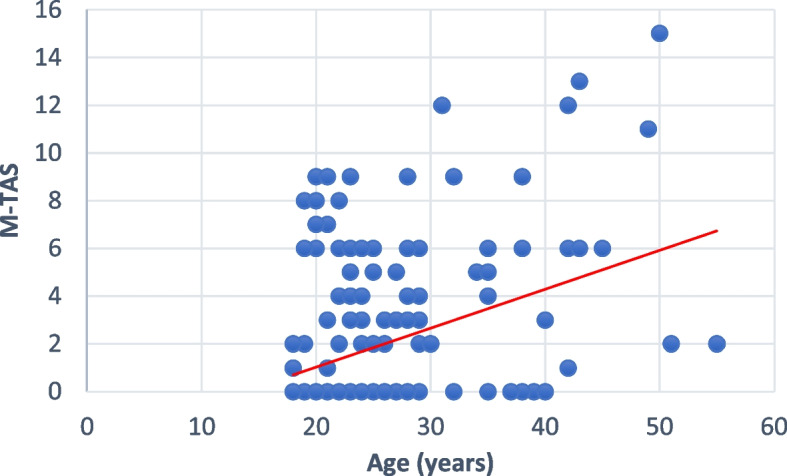
Scatter plot showing the relationship between the M-TAS and participants’ ages

The scatter plot representing the Marginal Traction Alopecia Severity Score (M-TAS) according to the participants’ age shows a moderate, positive and linear relationship between these two variables (Fig. 3). The bivariate correlation however showed a positive but weak relationship between age and the M-TAS (*r* = 0.235; *p* < 0.001). The equation was M-TAS = 0.148 (age) – 2.044.

### Multivariate analysis

On multivariate analysis, we found that women aged of 35 years and above were 4 times more susceptible to develop TA compared to women aged between 18 and 24 years (*p* = 0.016). Though the probability of TA reduced with the level of education, this result was not statistically significant. The profession was not associated to TA (Table 5).

**Table 5:** Sociodemographic characteristics of participants on multivariate analysis

Characteristics *N* = 223	TA *n* = 77 (%)	No TA *n* = 146 (%)	OR (95% CI)	*p*-value
**Age group**				
**18–24 years**	37 (48.1)	86 (58.9)	1	
**25–34 years**	23 (29.8)	49 (33.6)	1.3 (0.6–2.8)	0.484
**35–55 years**	**17 (22.1)**	**11 (7.5)**	**4.1 (1.3–12.9)**	**0.016**
**Level of education**				
**Primary**	8 (10.4)	3 (2.1)	1	
**Secondary**	16 (20.8)	15 (10.3)	**0.6 (0.1–3.1)**	0.503
**Higher**	53 (68.8)	128 (87.6)	**0.4 (0.1–2.3)**	0.316
**Profession**				
**Hairdresser**	9 (11.7)	7 (4.8)	**2.8 (0.5–14.5)**	0.214
**Others**	68 (88.3)	139 (95.2)	1	

Women whose hairstyles were exclusively done by a qualified person seemed less prone to have TA than those whose hairstyles were done both by a hairdresser and untrained persons (adjusted OR = 0.2; *p* = 0.008). Also, women that do not regularly wear nets, caps and head ties were 5 times less susceptible to develop TA (*p* = 0.006). The frequency of hairdressing related symptoms did not expose to TA (Table 6).

**Table 6:** Factors related with hair grooming habits and hair chemical treatment on multivariate analysis

**Factors related with hair grooming habits after multivariate analysis**				
**Characteristics** ***N* = 223**	**TA** ***n* = 77 (%)**	**No TA** ***n* = 146 (%)**	**OR (95% CI)**	***p*** **-value**
**Hair grooming provider**				
**Family member / friend who is not hairdresser**	6 (7.8)	17 (11.6)	0.3 (0.5–1.5)	0.138
**Hairdresser**	59 (76.6)	124 (84.9)	**0.2 (0-0.6)**	**0.008**
**Both**	12 (15.6)	5 (3.4)	**1**	
**Head tie/cap/net use**				
**No**	5 (6.5)	24 (16.4)	**0.2 (0-0.6)**	**0.006**
**Yes**	72 (93.5)	122 (83.6)	1	
**Hairdressing related symptoms**				
**Always**	8 (10.4)	12 (8.2)	1	
**Sometimes**	23 (29.9)	56 (38.4)	0.7 (0.2–2.5)	0.638
**Never/rarely**	46 (59.7)	78 (53.4)	0.5 (0.2–1.9)	0.340
**Factors related with chemical hair treatment after multivariate analysis**				
**Characteristics** ***N* = 223**	**TA** ***n* = 77 (%)**	**No TA** ***n* = 146 (%)**	**OR (95% CI)**	***p*-value**
**Age at first hair relaxation**				
**Before 10 years**	16 (20.8)	20 (13.7)	**0.8 (0.3–1.9)**	0.617
**After 10 years**	61 (79.2)	126 (86.3)	1	
**Hair relaxation/ year**				
**No more than once**	11 (14.3)	64 (43.8)	**0.2 (0.1–0.6)**	**0.005**
**2–3 times**	38 (49.3)	60 (41.1)	**0.4 (0.4-1.0)**	0.550
**>3 times**	28 (36.4)	22 (15.1)	**1**	
**Bruns after relaxation**				
**Never/rarely**	41 (53.2)	82 (56.2)	**0.6 (0.2–1.9)**	0.423
**Sometimes**	23 (30)	52 (35.6)	0.4 (0.1–1.3)	0.123
**Often/always**	13 (16.8)	12 (8.2)	1	

Women relaxing their hair once in a year or less frequently were 5 times less subject to TA than those who relaxed twice or thrice a year (adjusted OR = 0 2; *p* = 0.005). The age at the first hair chemical straightening was not associated to TA (Table 6).

## Discussion

TA is a common disease but still less studied particularly in Africa. The aim of our study was to research factors associated with TA occurrence in women living in Yaoundé. We found that factors associated with TA were: age, hair style provider, the use of head ties, caps and nets and the annual frequency of chemical hair relaxation.

Concerning the sociodemographic factors, we found that participants with TA were older than patients without TA. This result aligns with study by Dadzie and Salam in 2015 in United Kingdom [13].

An age of 35 years or above increased by 4 folds the probability of developing TA (*p* = 0.016). Hence, there was a positive correlation between age and M-TAS, though this correlation was weak. Age was also identified by Dadzie et al. (OR = 1.04 ; *p* < 0.001) [13]. In fact, hair reach their growth peak at the age of 30. After this age, there is a diminution of the hair shaft’s diameter and a reduction of hair density ; hairs also become dryer [14]. These modifications are exacerbated by the use of hair dye and the use of flat iron [15]. Given this fragility of hairs with age, reducing aggressive treatment on them (chemical straightening, hair dryer or heat iron use) could contribute to reduce the occurrence of TA.

Women that usually use extensions for their hair grooming develop TA more than women who did not use them. In fact, causality between TA and hairstyles that chronically put in tension the hair shaft (dreadlocks, wigs, braids) is established [8, 16]. Extensions are natural or synthetic additives used in order to increase the size or the length of the hair [17]. Though extensions make hairstyles daily maintenance easy, they impose an additional charge to afro hair that is relatively fragile and less extensive; this favouring hair breakage. Moreover, women of Afro-descent prefer hairstyles with extensions because they a be kept for longer periods [18].Yet, the weight and the duration of wearing of these extensions also favour TA occurrence [10]. Extensions also exert a permanent and one direction traction on hairs. This could lead to and important rupture of hair shafts when added to hairs initially fragilized [19, 20]. A measure to avoid TA could consist of sensitising women in order for them to reduce the length and the volume of extensions, as well as the duration of their hairstyles. Also, hairstyles without extensions should be proposed as alternative solution particularly to women above 35 years old.

We did not find an association between hairdressing related symptoms and TA like other studies reported [8, 10, 13]. This could be due to the fact that majority of women in the present study rarely had such symptoms during hairdressing or asked the hairdresser to loosen their braids.

Women whose hairstyles were done only by a hairdresser were 5 times less susceptible to develop TA compared to women whose hairstyles were done both by a hairdresser and an untrained person (*p* = 0.008). Dadzie and Salam found that women having their hair grooming undertaken by a friend / family member with formal hairdressing training and qualifications were at risk of developing Alopecia [13]. We can presume that subjects with effective hair loss feel more comfortable to have their hair grooming by a family member rather than visit a hairdressing saloon where privacy is not always present; the friend/family member may be trained but don’t have all the appropriate equipment; which can lead to a vicious circle. Therefore, the requirement of professional services (qualified person in a well-equipped milieu) for hair grooming could help preventing TA in women. Also, hairdressers should be more sensitised on TA and retrained on the haircare and manipulation of « afro » hair.

Regular use of caps, nets and head ties was more common in TA group compared to the group without TA. We found that avoiding such accessories reduced by 5 times the probability of TA (adjusted OR = 0.2; *p* = 0.006). The regular use of tied buns has already been presented in Literature as a factor influencing TA’s onset [8]. These accessories are generally worn tightly and exerting traction on hairs. Moreover, women using them have their hair done before wearing those accessories. Religious or cultural motivations could also justify the regular use of various beauty accessories; it is the case for Sikh religion adepts who wear a head tie (turban) constantly. They later on develop TA [6, 21]. Nevertheless, even if the role of caps in TA onset is not firmly established; few studies like the one of Billero and al. found that localized alopecia was present in seven nurses whom attached cap to the scalp [6]. Our study globally evaluated the frequent use of these accessories. It seems necessary and appropriate that further studies should be carried out on the subject to enlighten this problematic. However, it could be judicious to educate women on the adequate use of these accessories particularly head ties and nets which seem very used by afro descent women.

Hair relaxation was the main chemical hair treatment used by women presenting TA. A frequency of hair straightening equal to once a year or less could be a protective factor from TA (adjusted OR = 0.3; *p* = 0.025). Rucker et al. also found an increasing risk of TA with chemical hair relaxant’s use [22]. In fact, hair straightening products destroy the monomolecular layer of fatty acid covalently bound to the cuticle. This hydrophobic layer retards water from wetting and penetrating the hair shaft and changing its physical properties [23, 24]. Thus, the relaxed hair is porous and less resistant to traction than the natural « afro » hair [20]. The cysteine residues of relaxed hair shafts are reduced ; meanwhile cysteine is a component of disulfuric bonds that contribute to hair rigidity [25]. This result could also mean that it is long term damages caused by hair relaxation that are deleterious for hairs. Given that hair relaxation is combined to other haircare practices namely extensions use, the risk of TA is bigger [8, 10, 19]. Sensitisation on the deleterious effect of hair relaxers on hairs, « afro » hair promotion and valorisation campaigns through media and especially social media, women sensitization on the good practices and the norms of usage of hair relaxer and increasing taxes on hair relaxers are several measures to restrict relaxers use and lessen TA in these women.

The earliness of the first chemical treatment was not associated to TA in our study. To our knowledge, there is no study that determined the relation between early onset of hair straightening and TA. But we did not collect the exact age at which the first chemical hair relaxation was done. It was then difficult to exclude an association between these two entities. That is why further research needs to be done to better explore the relation between both entities.

### Limits of the study

Our study could present some limits, namely the fact that: *(i)-* Covid-19 pandemic considerably reduced our data collection period; *(ii)-* our study could present an information bias since data collected were relative to anterior exposures; *(iii)-* some data collected could be subjective; and *(iv)-* hairdressing saloons though chosen randomly, the sample obtained could not be representative of the town’s population. Nevertheless, this study is primer for further studies on the subject at a larger scale.

## Conclusion

Our study found an association between TA and sociodemographic indicators and haircare practices. Afro descend women (Black women) should be informed on the susceptibility to develop TA which increases with age. The value of « afro » hair must be restored to afro descent women. Women should be sensitised on the long-term noxious effects of some haircare practices and alternatives should be proposed to these women in order to adopt less aggressive haircare practices for their hair. Cosmetics use must be done respectfully of the norms and code of good practices. In order to better understand and prevent TA, further studies should be carried out to research other predictive factors of TA’s severity.

## Data Availability

All data relevant to the study are included in the article. No additional data available, all data relevant to the study are included in the article.

## References

[1] Marks DH, Penzi LR, Ibler E, Manatis-Lornell A, Hagigeorges D, Yasuda M (2019). The Medical and Psychosocial associations of Alopecia: recognizing hair loss as more than a Cosmetic concern. Am J Clin Dermatol.

[2] Horev L. Environmental and Cosmetic Factors in Hair Loss and Destruction. In: Tur E, éditeur. Current Problems in Dermatology. Basel: KARGER; 2007 [cité 30 nov 2019]. p. 103–17. Disponible sur: https://www.karger.com/Article/FullText/106418. 10.1159/000106418 17641493

[3] Alopécie. - Troubles dermatologiques. Édition professionnelle du Manuel MSD. [cité 28 nov 2019]. Disponible sur: https://www.msdmanuals.com/fr/professional/troubles-dermatologiques/troubles-des-cheveux-et-des-poils/alop%C3%A9cie.

[4] Kanti V, Röwert-Huber J, Vogt A, Blume‐Peytavi U (2018). Cicatricial alopecia. JDDG J Dtsch Dermatol Ges.

[5] Kluger N, Cavelier-Balloy B, Assouly P (2013). Les alopécies par traction. Ann Dermatol Vénéréologie avr.

[6] Billero V, Miteva M (2018). Traction alopecia: the root of the problem. Clin Cosmet Investig Dermatol.

[7] Tanus A, Oliveira CCC, Villarreal DJV, Sanchez FAV, Dias MFRG (2015). Black women’s hair: the main scalp dermatoses and aesthetic practices in women of African ethnicity. An Bras Dermatol.

[8] Salam A, Aryiku S, Dadzie OE. Hair and scalp disorders in women of African descent: an overview. Br J Dermatol 2013. 2013;169(3):19–32. 10.1111/bjd.12534 24098898

[9] Sheikh Zuhayr AA, Dina. Al Abadie Mohammed. the-impact-of-female-pattern-hair-loss-on-quality-of-life-ijced-19.pdf. 2019. 2019^e^ éd. United Kingdom; Disponible sur: https://www.opastonline.com.

[10] Mirmirani P, Khumalo NP (2014). Traction Alopecia. Dermatol Clin.

[11] Khumalo NP, Jessop S, Gumedze F, Ehrlich R (2008). Determinants of marginal traction alopecia in African girls and women. J Am Acad Dermatol.

[12] Khumalo NP, Ngwanya RM, Jessop S, Gumedze F, Ehrlich R. Marginal traction alopecia severity score: development and test of reliability: marginal traction alopecia severity score. J Cosmet Dermatol. 2007;30(4):262–9. 10.1111/j.1473-2165.2007.00345.x 18047612

[13] Dadzie OE, Salam A. Correlates of hair loss in adult women of African descent in London, U.K.: findings of a cross-sectional study. Br J Dermatol. 2015;173(5):1301–4. 10.1111/bjd.13917 25998579

[14] Fernandez-Flores A, Saeb‐Lima M, Cassarino DS (2019). Histopathology of aging of the hair follicle. J Cutan Pathol Juill.

[15] Turner GA, Bhogal RK (2016). Hair and Aging Skinmed.

[16] Lawson CN, Hollinger J, Sethi S, Rodney I, Sarkar R, Dlova N (2017). Updates in the understanding and treatments of skin & hair disorders in women of color. Int J Womens Dermatol.

[17] Hair extension | signification, définition dans le dictionnaire Anglais de Cambridge. [cité 1 août 2020]. Disponible sur: https://dictionary.cambridge.org/fr/dictionnaire/anglais/hair-extension.

[18] Hall RR, Francis S, Whitt-Glover M, Loftin-Bell K, Swett K, McMichael AJ (2013). Hair Care practices as a barrier to physical activity in African American women. JAMA Dermatol.

[19] Haskin A, Aguh C (2016). All hairstyles are not created equal: what the dermatologist needs to know about black hairstyling practices and the risk of traction alopecia (TA). J Am Acad Dermatol.

[20] Aryiku SA, Salam A, Dadzie OE, Jablonski NG (2015). Clinical and anthropological perspectives on chemical relaxing of afro-textured hair. J Eur Acad Dermatol Venereol.

[21] Gupta D, Thappa DM (2015). Dermatoses due to Indian Cultural practices. Indian J Dermatol.

[22] Rucker Wright D, Gathers R, Kapke A, Johnson D, Joseph CLM (2011). Hair care practices and their association with scalp and hair disorders in African American girls. J Am Acad Dermatol.

[23] Quaresma MV, Martinez Velasco MA, Tosti A (2015). Hair breakage in patients of African descent: role of Dermoscopy. Skin Appendage Disord.

[24] Essel EA, Ahenkorah J, Blay RM, Adjenti SK, Adutwum-Ofosu KK, Hottor BA et al. Microscopic characteristics of scalp hair subjected to Cultural Styling methods in Ghanaian African females. Clin Cosmet Investig Dermatol. 2019;12:843–50. 10.2147/CCID.S225627 PMC6873963 31819581

[25] Ogunleye TA, McMichael A, Olsen EA (2014). Central Centrifugal Cicatricial Alopecia. Dermatol Clin.

